# Density-Dependent Growth and Fitness in *Dastarcus helophoroides* (Coleoptera: Bothrideridae)

**DOI:** 10.3390/insects10110386

**Published:** 2019-11-04

**Authors:** Shang-kun Gao, Cui-cui Geng, Ying-chao Ji, Zi-kun Li, Cheng-gang Zhou

**Affiliations:** 1 Shandong Research Center for Forestry Harmful Biological Control Engineering and Technology, College of Plant Protection, Shandong Agricultural University, Tai’an 271018, China; skgao@sdau.edu.cn (S.-k.G.); yingchao0402@163.com (Y.-c.J.); fclzk123@163.com (Z.-k.L.); 2 Taishan Food and Drug Testing Center, Shandong Medical Technician College, Tai’an 271000, China; gengcuicui01@126.com

**Keywords:** *Dastarcus helophoroides*, larva, density, developmental performance, adult fitness, natural enemy insect

## Abstract

The ectoparasitoid *Dastarcus helophoroides* Fairmaire (Coleoptera: Bothrideridae) is an important natural enemy insect, which is artificially mass-reared and released into woodland to control medium and large longhorn beetle species. This study examined the developmental duration (days) of larvae and adult fitness (including numbers of adults emerging per host and mean body size) by exposing a single substitute host, a pupa of *Zophobas morio* (Coleoptera: Tenebrionidae), to different densities of *D. helophoroides* larvae. We showed that there was no significant effect on the rate of successful parasitism and cocoon formation, but emergence success and measures of individual adult body size (length, width, and weight) declined with increasing larval density. Larval period and cocoon period increased with larval density, while total weight of adults emerging per host increased initially before reaching a plateau. Our results suggest that a pupa of *Z. morio* could be successfully parasitized by a single *D. helophoroides* larva, but multiple *D. helophoroides* larvae can share one host. Excessive larval density caused intraspecific competition among *D. helophoroides* larvae, manifesting in extended developmental duration of immature stage and reduced fitness of adults. Furthermore, the tradeoff between the numbers of adults and body size may stabilize the population dynamics with detectable mutual interference, particularly in competing for limited host resources. These findings suggest six larvae per host would achieve the highest adult fitness and would enhance mass-rearing techniques as part of IPM strategies for longhorn beetles.

## 1. Introduction

In the past two decades, an increase in artificial large plantings of forest monocultures has increased the detrimental effects of beetle pests, especially those of longhorn beetles. Global warming has caused the extension of their distribution range and impact [1,2,3]. For example, Poplar trees planted in Three North Shelterbelt in China have been seriously damaged by the Asian longhorn beetle *Anoplophora glabripennis* Motschulsky (Coleoptera: Cerambycidae), causing an estimated annual loss of approximately $1.5 billion [4]. A similar species, *A. chinensis* Forster, has also caused severe damage to citrus trees and pure casuarina forests in China and has been listed as an important quarantine species in Europe and North America [5,6]. The pine longhorn beetle *Monochamus alternatus* Hope (Coleoptera: Cerambycidae) is one of the most dangerous borer pests in China, as it spreads pinewood nematode disease (PWN) via adult supplemental nutrient and oviposition wounds, causing the deaths of large areas of pine forests in a short time [7]. The epidemic areas of PWN have risen to 18 provinces since it was first discovered in 1982, causing more than fifty million pine trees to die and economic losses of more than twenty-five billion RMB in China. Most of the time longhorn beetles are hidden inside tree trunks, which makes traditional control measures (e.g., chemical insecticides) ineffective.

So far, the ectoparasitic beetle *Dastarcus helophoroides* Fairmaire (Coleoptera: Bothrideridae) is the most effective parasitoid against various medium and large wood-boring insects [8], such as *A. glabripennis*, *A. chinensis*, *Massicus raddei* (Coleoptera: Cerambycidae), *M. alternatus*, *Batocera horsfieldi* (Coleoptera: Cerambycidae), *Apriona germari* (Coleoptera: Cerambycidae), *A. swainsoni*, and *Xylocopa appendiculata* (Hymenoptera: Apidae). *Dastarcus helophoroides* has been found in China and Japan [9,10]. Eggs are laid near the host entrance hole or frass-extrusion hole twice per year by adults in the wild [9,11]. Once the eggs hatch, first instar larvae with well-developed pectoral feet actively forage for a suitable host and their feet do not degenerate until they have successfully parasitized a host. The developmental duration of egg, larval and pupal periods are 12.7, 8.4, 25.6 days at 21 ± 1 °C, respectively [11], and the life expectancy of adults is over three years [8]. It is difficult to distinguish male and female adults by external morphological characteristics, but examination of the end angle of the anal plate and the length and width of wing and its trigonal end zone, allow identification of sex with 70–80% accuracy [12].

Mass-rearing of insect natural enemies is an important basis for the successful biological control of pests, which is an important strategy in an integrated pest management (IPM) program. The beetle *D. helophoroides* is a parasitic Coleopteran natural enemy insect that has a long lifespan (3 to 6 years) and oviposition period (ovipositional diapause has been not found so far), and a harder exoskeleton than most parasitic Hymenoptera enemy insects [8,13]. Greater numbers of *D. helophoroides* eggs can be produced by artificial feeding of adults, improves parasitic efficiency in substitute hosts [3].

In recent years, there have been many studies on mass-rearing, alternative host selection, and the factors affecting parasitism rates by *D. helophoroides* in China [14,15,16]. It has been artificially reared in large numbers and inoculatively or inundatively released into forests to control longhorn beetle pests [17,18,19,20,21,22]. These reports have proved that *D. helophoroides* is an important natural enemy insect parasitizing longhorn beetles in China. However, there are many differences between this parasitic beetle and most hymenopteran parasitoids in terms of patch exploitation strategies, life-history traits, ontogeny strategies, and intraspecific competition. The main difference is that the parasitic process is completed by adult females in Hymenoptera parasitoids, while the parasitic stage of *D. helophoroides* is the larvae. Similar parasitic modes also occur in other orders of holometabolous insects: Diptera, Lepidoptera, and Neuroptera [23,24]. For example, host-seeking behavior [25], ontogeny [26], life-history traits [27], spatial density dependence [28] and intraspecific competition [29] of the parasitic robber fly *Mallophora ruficauda* (Diptera: Asilidae) have been reported in detail. In the ‘several parasitoids–one host’ system, larvae/egg density inside or external to the host is determined by females checking multiple hosts before choosing the ones with the highest fitness potential [30,31,32]. The inoculated larvae of *D. helophoroides* require a host that has not been paralyzed or killed by an adult. This differs from the larvae of idiobiont wasps that feed directly within the host that has been paralyzed or killed by an adult or adults [33].

As a new protein source insect introduced from southeast Asian countries, *Zophobas morio* Fabricius (Coleoptera: Tenebrionidae) is mass-reared and mainly used as living bait in China [34]. Currently, Pupae have been used as high-quality substitute hosts for artificially mass-reared *D. helophoroides* in our laboratory. Inoculation density is one of the main factors affecting parasitism adaptability and seriously restricts the efficiency of artificial mass-rearing of parasitic enemy insects [31]. The tradeoff between the development of offspring and host quality (e.g., body size, host nutrition) among adults in some Hymenoptera parasitoids has already been proved [31,32,35]. However, questions still remain regarding the interaction between *D. helophoraides* larvae and their host such as: (1) Is the rate of successful parasitism related to larval density? (2) Is ontogenesis of parasitic larvae affected by intraspecific competition at high densities? (3) When the density of parasitic larvae is increased, is there a trade-off between numbers of adults emerging per host and the individual fitness of those adults, as occurs in female parasitic wasps.

In this study, *D. helophoroides* larvae with various densities were inoculated into a single pupa of *Z. morio*. The objective of our present study was to examine the effect of larval densities on developmental durations of larvae and fitness of emerging adults. We compared parameters such as larval period, cocoon period, body size (length and width) and the bodyweight of a single adult, total weight and numbers of adults emerging from one host at various larval densities. Finally, we relate our predictions concerning an optimal larval density to mass-rearing *D. helophoroides*, depending on host availability and competition among larvae.

## 2. Materials and Methods

### 2.1. Experimental Materials

We established a laboratory population of *D. helophoroides* from adults taken from the Research Institute of Forest Ecology, Environment, and Protection, Chinese Academy of Forestry. The adults were maintained on an artificial diet for 2 years at 25 ± 1 °C and 60–70% RH under an LD 8:16 h light regime. Brown paper with eggs was collected twice weekly and kept in a 25 °C light regime. Newly hatched larvae were selected for the inoculation experiment.

The 3–4 cm larvae of *Z. morio* were purchased from a farm named Happy Farmers in Quzhou City, Zhejiang Province. When larvae became inactive, they were collected and put into pupation boxes at a density of 500 g per box (40 × 25 × 10 cm). We monitored the changes from larvae to pupae daily. Host pupae were kept in a 5 °C refrigerator and were weighed using an analytical balance (sensitivity 0.1 mg). 120 pupae (weight range 550.0–600.0 mg) were selected for our experiment. Although there was a 50 mg variation among the hosts used, our preliminary test indicated that the interaction between host weight and larval density was not significant [31,35]. We regarded host size as a constant.

### 2.2. Inoculation of Dastarcus helophoroides Larvae

Individual hosts were exposed to different numbers of newly hatched *D. helophoroides* larvae (from one to ten) in a flat plastic tube (42 × 13 mm). A piece of folded brown paper was inserted into the tube and then the opening of each tube was blocked using a cotton plug. We established 30 tubes for each of the 10 densities examined. All experimental insects were maintained in an artificial climate chamber at 25 ± 1 °C and 60–70% RH under an LD 8:16 h light regime.

### 2.3. Observation of Dastarcus helophoroides Larvae Performance

We monitored the developmental processes of *D. helophoroides* larvae for each replicate vial. We defined the larval period (days) as the period between the inoculation date and cocoon formation date. We defined the cocoon period (days) as the period from cocoon formation date to adult emergence date. Then, we calculated both the cocoon formation rates and emergence rates. We observed all parasitoid hosts daily under a microscope.

### 2.4. Determination of Dastarcus helophoroides Adult Fitness

The body length and width of each newly emerged adult were measured using Vernier calipers (sensitivity of 0.1 mm). Body size was measured as the length from the head to the tip of the elytra and the width between the bases of the two elytra. Each adult was weighed using an analytical balance (sensitivity 0.1 mg). The numbers and total weight of adults emerging per host were recorded. Adult fitness was estimated using the bodyweight of each *D. helophoroides* adult.

### 2.5. Statistical Analysis

All data were analyzed using GraphPad Prism version 5.0 for Windows (Graph-Pad Software, San Diego, CA, USA). We assessed that the relationships between larval densities and parasitic parameters (developmental performance of *D. helophoroides* larvae: larval period, cocoon period; adult fitness: body length, body width, individual weight, total weight). We performed a Kruskal-Wallis test to compare differences in the rate of successful parasitism, the proportion of cocoon formation, rate of successful emergence and numbers of emerging adults per host. Furthermore, we used one-way analysis of variance (ANOVA) to assess the differences among treatments in larval period, cocoon period, body length, body width, and weight of a single adult, total weight of adults emerging per host. Regression analyses were used to describe the relationships between parasitic parameters (body length, body width and weight of a single adult, total weight of adults emerging per host) and larval densities.

## 3. Results

### 3.1. Developmental Performance of D. helophoroides Larvae with Various Densities

There were no significant differences in the rate of successful parasitism among treatments (*n* = 12, *H* = 6.207, *p* = 0.719) with an overall mean value of 96.7% (Table 1). Treatment had a significant effect on the larval period of *D. helophoroides* (*F* = 32.615, *df* = 9, 106, *p* = 0.0001) with values ranging from 8.42 d (one larva per host) to 12.53 d (ten larvae per host) (Table 1). The proportion of *D. helophoroides* larvae forming cocoons was not significantly influenced by treatment (*n* = 12, *H* = 11.202, *p* = 0.262) with an overall mean value of 88.7% (Table 1). The cocoon period significantly increased with increasing larval density (*F* = 11.926, *df* = 9, 93, *p* = 0.0001) with value ranging from 25.92 d (one larva per host) to 36.66 d (ten larvae per host) (Table 1). There was a significant effect of larval density on the rate of successful emergence (*n* = 104, *H* = 18.63, *p* =0.029) with values ranging from 73.81% (ten larvae per host) to 100% (both one larva per host and three larvae per host). The rates of successful emergence of *D. helophoroides* adults decreased with increasing larval density (Table 1).

### 3.2. Adult Fitness

**Figure 1:**
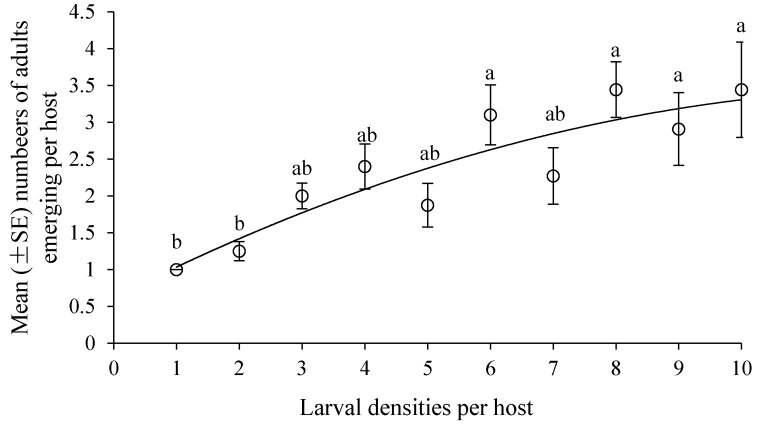
Mean (± SE) numbers of adults emerging per host with various larval densities (regression: *y* = −0.0166*x*^2^ + 0.435*x* + 0.6155; adjusted *r*² = 0.8127, *p* = 0.0001). The different letters above each larval density show significant difference among treatments at a = 0.05.

The mean number of adults emerging per host differed significantly among the treatments (*n* = 104, *H* = 43.43, *p* < 0.0001, Figure 1). The mean number of adults emerging per host increased with increasing larval densities per host. Significantly more were found in vials with one host exposed to 6, 8, 9 and 10 larvae than in vials with one host exposed to 1 and 2 larvae (*n* = 63, *H* = 32.94, *p* < 0.0001).

**Figure 2:**
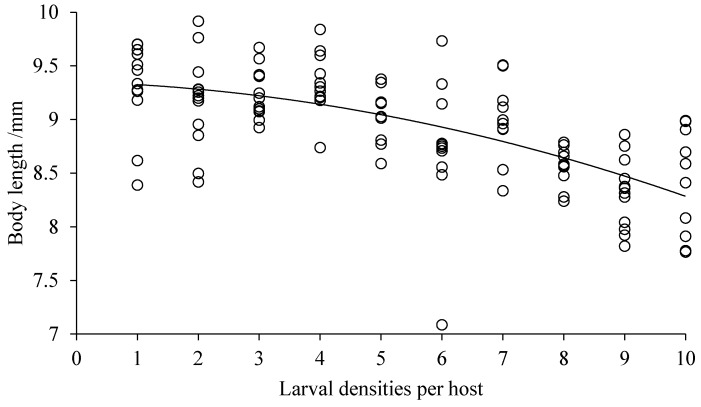
Body length of *D. helophoroides* adults emerging per host with various larval densities. Relationship between larval densities per host and body length width of *D. helophoroides* adults (*y* = −0.0091*x*^2^ − 0.0153*x* + 9.3497; adjusted *r*² = 0.8655, *p* = 0.0001).

**Figure 3:**
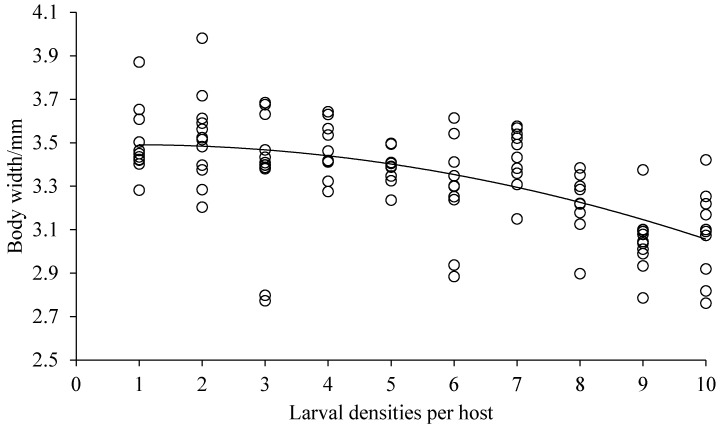
Body width of *D. helophoroides* adults emerging per host with various larval densities. Relationship between larval densities per host and body width of *D. helophoroides* adults (*y* = −0.0052*x*^2^ + 0.009*x* + 3.4871; adjusted *r*² = 0.8499, *p* = 0.0001).

**Figure 4:**
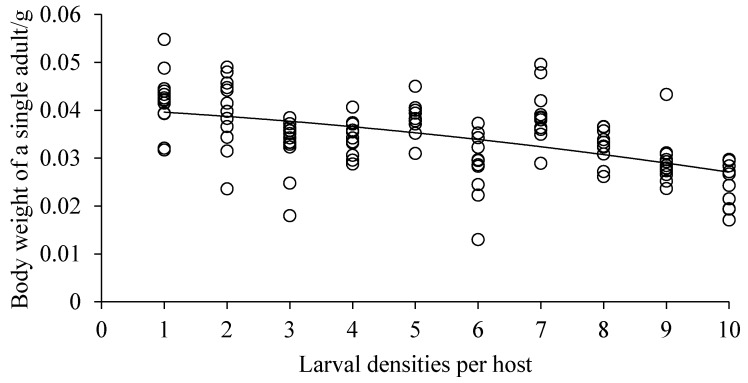
Bodyweight of a single *D. helophoroides* adult with various larval densities. Relationship between larval densities per host and bodyweight of a single *D. helophoroides* adult (*y* = −0.00006*x*^2^ − 0.0007*x* + 0.0404; adjusted *r*² = 0.5594, *p* < 0.01).

**Figure 5:**
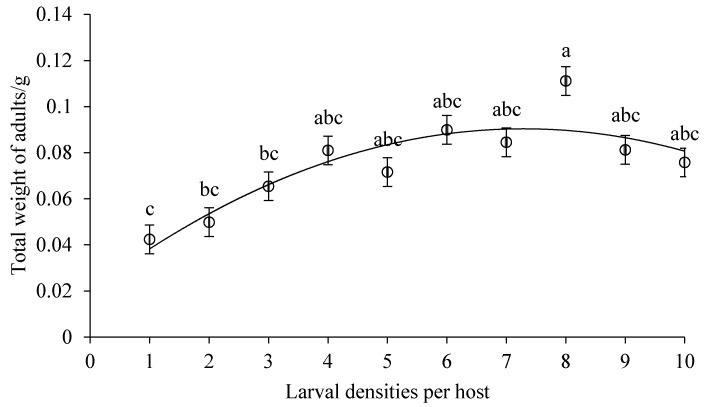
The total weight of adults emerging per host with various larval densities. Relationship between larval densities per host and the total weight of adults emerging per host (*y* = −0.0013*x*^2^ + 0.0192*x* + 0.0203; adjusted *r*² = 0.7874, *p* < 0.01). See Figure 1 legend for interpretation.

Both the body length and width of *D. helophoroides* adults emerging from cocoons decreased significantly with the increased larval densities (body length, *F* = 9.144, *df* = 9, 92, *p* = 0.0001, Figure 2; body width, *F* = 7.788, *df* = 9, 93, *p* = 0.0001, Figure 3). Hosts that were parasitized by more *D. helophoroides* larvae bred smaller adults. The bodyweight of a single *D. helophoroides* adult decreased as the larval densities increased (*F* = 10.081, *df* = 9, 93, *p* = 0.0001; Figure 4), according to a significant quadratic term (*p* < 0.01). The mean total weight of adults successfully emerging per host increased with larval densities up to a larval density of five per host (Figure 5). Above larval densities of five, the average total weight of adults emerging per host remained similar, though variability around the mean dramatically decreased.

## 4. Discussion

Various parasitic larvae–host interaction was investigated in our study. The results showed that there was no significant difference in the rate of successful parasitism and cocoon formation among treatments, but emergence success and measures of individual body size (length, width, and weight) declined with increasing larval density (Table 1, Figure 2, Figure 3 and Figure 4). The duration of larval period and cocoon period were positively related to larval density (Table 1), while the total weight of adults emerging per host increased at low larval densities before reaching a plateau at higher densities (Figure 5). The body size of the selected host, a pupa of *Z. morio*, was fixed, and we surmised, therefore, that the density-dependent parasitism effects were mainly caused by intraspecific competition among larvae under conditions of a limited host resource.

The outcome of larval competition has been examined in tachinid species [36] and robber flies [28], including scramble competition, contest competition or both. The forms of larval competition depend on the utilization of resources by competing larvae [37]. Our results indicated that a single larva of *D. helophoroides* could parasitize a substitutive host pupa weighting 0.55–0.6 g with a 100% success rate. This is significantly higher than the 83.3% success rate observed for the mature larvae of *Monochamus alternatus* weighing 0.5–0.9 g [38]. As larval densities increased, two or more larvae can share a single host with 96.67% average successful parasitic rate, but not quasi-social phenomenon found in *Sclerodermus* spp. with higher parasitism rates and host utilization [31,39]. All the life-history parameters (growth and adult fitness) did not differ significantly when exposing one substitutive host to one and two larvae. We, therefore, conclude that scramble competition was not likely in competing larvae until beyond two larvae per host. When more than two *D. helophoroides* larvae per host were inoculated, competition for resources was more evident. Numbers (Figure 1) and total weight of adults emerging per host (Figure 5) remained constant up to larval densities of six and then kept steady, even declined slightly, suggesting that scramble competition may give way to contest competition at higher larval densities. Furthermore, adult size (body length, width, and weight of a single adult) declined rapidly as the larval densities exceeded six, which was indicative of contest competition.

In many parasitoid wasps, competition among larvae is initiated by the foundress after host evaluation of the host resources to regulate the progeny ratio [40,41,42]. However, in our experiment, intraspecific competition among *D. helophoroides* larvae was manipulated by artificial inoculation of densities ranging from 1 to 10 larvae per host. Our results indicated that larval periods were significantly extended as the larval density increased, in response to the limited host resources. When more than three larvae per host were introduced, the limited host resources initiated intraspecific competition among larvae and prolonged the larval period from less than 10 d to around 12 d. For pupal parasitoids, dramatic morphological and physiological changes (such as wings, appendages, and antennae) could arise with host age and inhibit the rate of consumption and digestion by the parasitoid larvae [43]. Therefore, another reason for the increase in larval periods may have been temporal changes in the fitness of the host pupae (pupae of *Z. morio* in the study).

Intraspecific competition always results in fitness reduction or the death of competing individuals [44], but there were no significant differences in the proportion of larvae forming cocoons, which indicated that host nutrition was adequate to facilitate cocoon formation by the surviving larvae. However, the cocoon period extended, and the rate of successful emergence declined as the larval density increased. Hence, in order to shorten the breeding time and improve the adult eclosion rate, we suggest that less than three *D. helophoroides* larvae are exposed to each host at most.

Although the developmental duration is an important life-history characteristic used to measure the performance of the parasitic larva, *D. helophoroides* larvae are ectoparasitoids insect and have greater plasticity than endoparasitoid insects [45]. It is generally recognized that body size (or bodyweight) of an adult is an important fitness-related feature [46,47]. Therefore, we tend to take the fitness of emerged adults as an important index to measure and evaluate the effect of larval density during the mass-rearing of *D. helophoroides*.

The fitness value of some traits and host exploitation patterns in parasitoids are important to understand the evolution of development strategies. For parasitic natural enemy insects, the development of the whole immature stage is completed in a single host so that host quality largely affects their developmental performance [48]. For gregarious parasitoids, when host size is fixed, larvae must compete for resources during development [49]. So, in this study, adult fitness was determined largely by the impacts of intraspecific competition on the development of *D. helophoroides* larvae. The result was consistent with *Homotrixa alleni* (Diptera: Tachinidae) larvae [36]. Life history evolution is subject to tradeoffs: when individuals benefit from a change in one life history feature, they pay a price for a corresponding change in another [50]. One of the essential questions in evolutionary ecology is to explore which life-history characteristics have tradeoffs influencing biological fitness [51]. Our results showed that there was a negative correlation between the numbers of adults emerging per host and adult body size when less than six larvae per host were introduced. It indicated that there was a tradeoff between the numbers of adults and their body size for utilizing limited host resources, as larval densities increased. However, when more than five larvae per host were introduced, there was no significant difference in the number of adults emerging per host (about three adults emerging per host or total weight of adults). The study indicated that a single host of 0.55–0.6 g can satisfy the growth and development of about five *D. helophoroides* larvae, and the fitness of the adults emerging per host remained unchanged. If the count was exceeded, to ensure the population quantity (mean numbers: three adults), the mean individual size of *D. helophoroides* larvae would be reduced. Hence, if the numbers, individual size and total weight of adults emerging per host are taken into account, we suggest that six *D. helophoroides* larvae per host is the most optimal.

Intraspecific larval competition has a significant effect on many aspects of adult fitness [49] and the dynamics and stability of their populations [36], especially for gregarious parasitoids. However, other adult fitness measures that were not determined in the study still need further research, such as the proportion of female adults, lifespan, fecundity, and reproductive strategy. Inundative release of *D. helophoroides* against longhorn beetles depend on their efficient mass-rearing. Excessive larval densities were found to be unbeneficial for improving efficiency of *D. helophoroides* mass-rearing. The results of our study highlight the importance of utilizing an optimal larval density in mass-rearing. Our results indicate that six larvae per substitutive host may achieve the highest adult fitness. We suggest that the release density of *D. helophoroides* should be adjusted according to the body size of target long-horn beetles, to help deliver an enhanced IPM strategy for longhorn beetles in China and Japan.

## Figures and Tables

**Table 1:** The developmental performance of *D. helophoroides* larvae with various densities. Data in the table refer to mean ±SE. Different letters within a column indicate significant differences among treatments (*p* < 0.05).

Host: Parasitic Larvae	n	Rate of Successful Parasitism	Larval Period (d) ± SE	Proportion of Larvae Forming Cocoon	Cocoon Period (d) ± SE	Rate of Successful Emergence
1:1	12	12/12	8.42 ± 0.15 c	12/12	25.92 ± 0.54 e	12/12 a
1:2	12	12/12	9.38 ± 0.30 c	12/12	29.79 ± 1.04 cde	15/16 a
1:3	12	12/12	11.81 ± 0.22 ab	12/12	29.65 ± 0.94 de	24/24 a
1:4	12	11/12	11.43 ± 0.24 b	10/11	29.77 ± 1.14 cde	24/26 ab
1:5	12	11/12	11.56 ± 0.32 ab	8/11	34.17 ± 1.45 abc	15/18 ab
1:6	12	11/12	12.43 ± 0.22 ab	10/11	35.04 ± 0.87 ab	31/34 a
1:7	12	11/12	11.25 ± 0.17 ab	9/11	35.22 ± 0.75 ab	25/30 ab
1:8	12	12/12	11.95 ± 0.12 ab	9/12	31.87 ± 0.59 bcd	31/37 ab
1:9	12	12/12	12.30 ± 0.23 ab	11/12	33.33 ± 0.78 abcd	32/33 a
1:10	12	12/12	12.53 ± 0.32 a	9/12	36.66 ± 1.54 a	31/42 b
